# 

**Published:** 1891-03

**Authors:** 


### Mississippi Valley Dental Association.

The forty-seventh annual meeting of the Mississippi Valley Dental Association will be held in Lincoln Club Hall, Cincinnati, March 10th, 11th, and 12th, 1891. The following programme has been arranged for:

President's address, Dr. M. H. Fletcher, Cincinnati; "Dentistry Thirty Years Ago and Now," Dr. J. W. Jay, Richmond, Ind; "Surgery of Cleft Palate," Dr. B. M. Rickets, Cincinnati; "Traumatic Lesions and Secondary Inflammation," Dr. F. W. Sage, Cincinnati; "Recent Advances in Dental Mechanism and Art," Dr. C. S. Smith, Chicago; "The Proper Sphere of the Dentist," Dr. H. H. Harrison, Wheeling; "Scientific Investigation of the Jaws and Cranium," Dr. Eugene S. Talbot, Chicago; "Why Copper Amalgam Sometimes Washes in the Mouth," Dr. W. B. Ames, Chicago; "Continuous Gum Clinic," Dr. George R. Riddell, Chicago.

There are other features that will be added to the above programme that will be announced later. The committee is putting forth every effort to make it an interesting meeting.

### THE DENTAL REGISTER,

A MONTHLY MAGAZINE DEVOTED TO THE INTERESTS OF THE

### DENTAL PROFESSION.

*Terms Reduced to $2.00 Per Year. Single Copies, 25 Cents.*

### EDITED BY PROF. J. TAFT, D.D.S.

—AND—

### Published by SAM'L A. CROCKER & CO., Ohio Dental and Surgical Depot.

The volume commences in January and closes in December.

The REGISTER being issued monthly is always fresh, therefore Indispensable to the practitioner who desires to keep posted up with the new inventions and improvements which are daily developed. Neither expense nor effort will be spared to give value and variety to its contents Articles will be illustrated with well executed engravings whenever they will add to the interest or assist to explanation.

Its extensive circulation and frequency of issue make it one of the BEST DENTAL ADVERTISING MEDIUMS in the United States.

All subscriptions must begin with January or July.

Local or special notices, five lines or less, will be inserted for $3 each insertion; over five lines, 50 cts. per line.

All advertising bills payable in advance, or at furthest, after the issue of the first number containing the advertisement. All transient advertisements to be paid for in advance.

☞CONTRIBUTIONS SOLICITED.

Exclusively Editorial Communications should be addressed to the Editor.

The latest number of the REGISTER may be ordered with or without other goods at any time, and all letters should be addressed to

SAM'L A. CROCKER & CO., Ohio Dental and Surgical Depot, Cincinnati, O.

